# Combined anatomical and functional diagnostic work-up of patients with suspected coronary artery disease using cardiac computed tomography and magnetic resonance imaging

**DOI:** 10.1186/1532-429X-13-S1-P77

**Published:** 2011-02-02

**Authors:** Jan GJ Groothuis, Aernout M Beek, Stijn L Brinckman, Martijn R Meijerink, Mijntje LP van den Oever, Mark BM Hofman, Cornelis van Kuijk, Albert C van Rossum

**Affiliations:** 1 VU University Medical Center, Amsterdam, Netherlands

## Introduction

The combined use of cardiac computed tomography coronary angiography (CTCA) and myocardial perfusion imaging allows the non-invasive evaluation of coronary morphology and function. Cardiovascular magnetic resonance imaging (CMR) has several advantages: it can simultaneous assess myocardial perfusion, ventricular and valvular function, cardiomyopathy and aortic disease and does not involve any ionizing radiation.

## Purpose

To investigate the usefulness of a combined non-invasive anatomical and functional work-up using cardiac computed tomography (CT) and CMR for the diagnostic evaluation of patients with suspected coronary artery disease (CAD) in clinical practice.

## Methods

Patients with low or intermediate pre-test probability of CAD underwent CT (Siemens Sensation 64, coronary calcium scoring and coronary angiography (CTCA)) and CMR (Siemens Sonata/Avanto, assessment of ventricular function, adenosine stress myocardial perfusion and late gadolinium enhancement imaging). Patients were referred for invasive coronary angiography (ICA) on the basis of obstructive CAD on CTCA (at least 1 lesion causing > 50% diameter stenosis), myocardial ischemia on CMR or clinical assessment by their physician. Fractional flow reserve measurements (FFR) were performed in case of intermediate lesions on ICA. Patients with no or non-obstructive CAD on CTCA and normal myocardial perfusion on CMR who did not undergo ICA were followed-up. The diagnostic performance for detection of significant CAD was determined for CTCA, CMR and the combination of CTCA and CMR. Significant CAD was defined as a FFR of ≤ 0.75. When no FFR measurements were available, a lesion was regarded as significant when the diameter stenosis was > 50% in two orthogonal directions using quantitative coronary analysis. Additional cardiac and extra-cardiac findings by CT and CMR were registered.

## Results

A total of 192 patients were included, 88 (46%) of whom underwent ICA. The combination of CTCA and CMR resulted in a significant improvement of specificity and overall accuracy for detection of significant CAD compared to CTCA or CMR alone: 94% and 91% versus 39% and 57%, p<0.0001 (CTCA) or 82% and 83%, p=0.016 (CMR), respectively. No events were recorded during follow-up (538±169 days) in 104 patients who did not undergo ICA. The combined strategy provided an alternative diagnosis in 18 patients (myocarditis, hypertrophic cardiomyopathy and dilated cardiomyopathy).

## Conclusions

The combined use of CT and CMR significantly improved the specificity and overall diagnostic accuracy for detection of hemodynamically significant CAD, and allowed the detection of alternative diagnoses.

